# Supplementary material for: The coenzyme thiamine diphosphate displays a daily rhythm in the Arabidopsis nucleus
Source: Commun Biol. 2020 May 5;3:209. doi: 10.1038/s42003-020-0927-z (PMC7200797; doi:10.1038/s42003-020-0927-z)
Supplement: Supplementary file 1 — Supplementary Information [file 42003_2020_927_MOESM1_ESM.pdf]

Supplementary Figures

Supplementary Figure 1a

thiC mutant

thiC 1.5 μM thiamine:*thiC* no thiamine

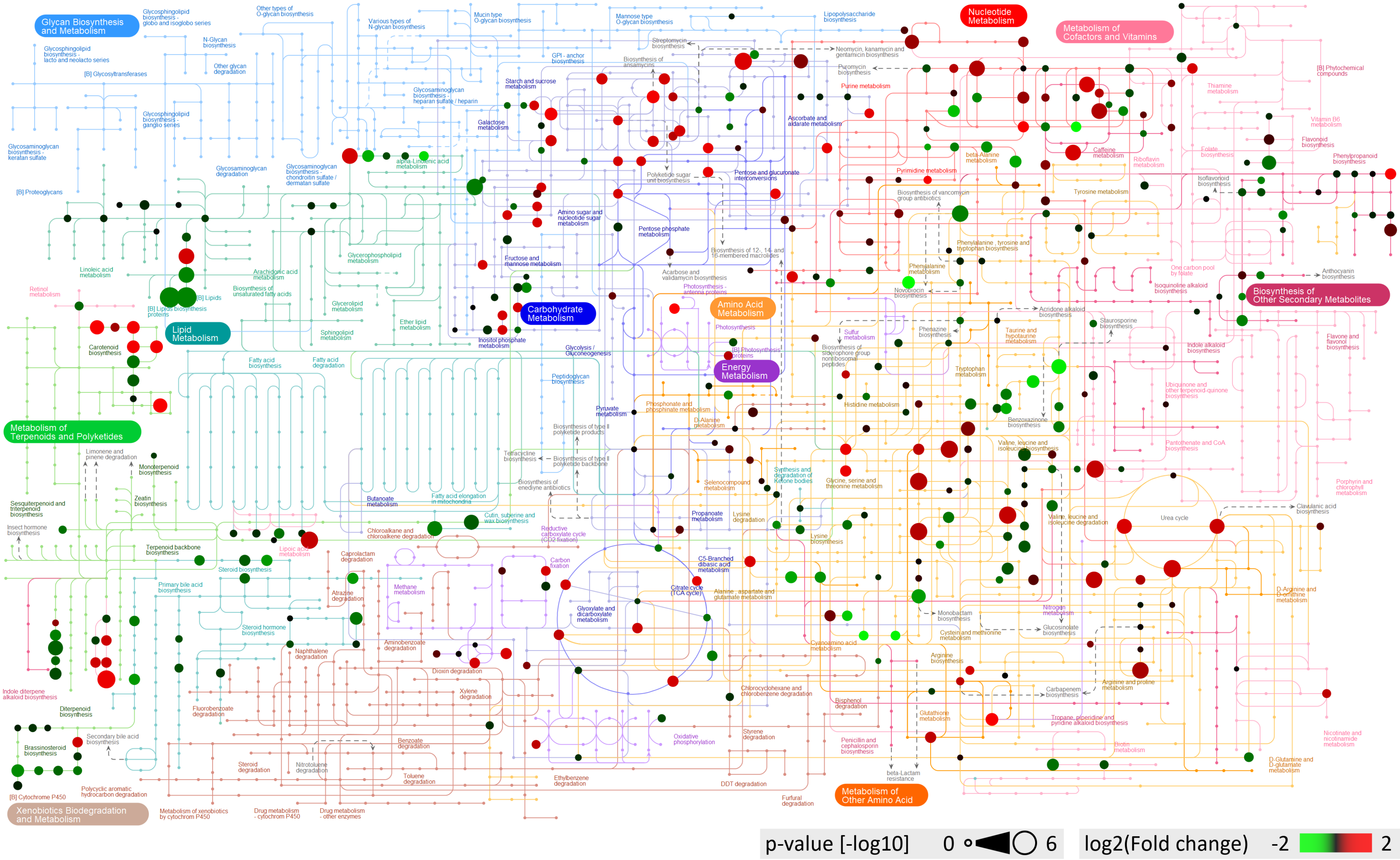

Supplementary Figure 1b

Wild type
Col-0 1.5 μM thiamine:Col-0 no thiamine

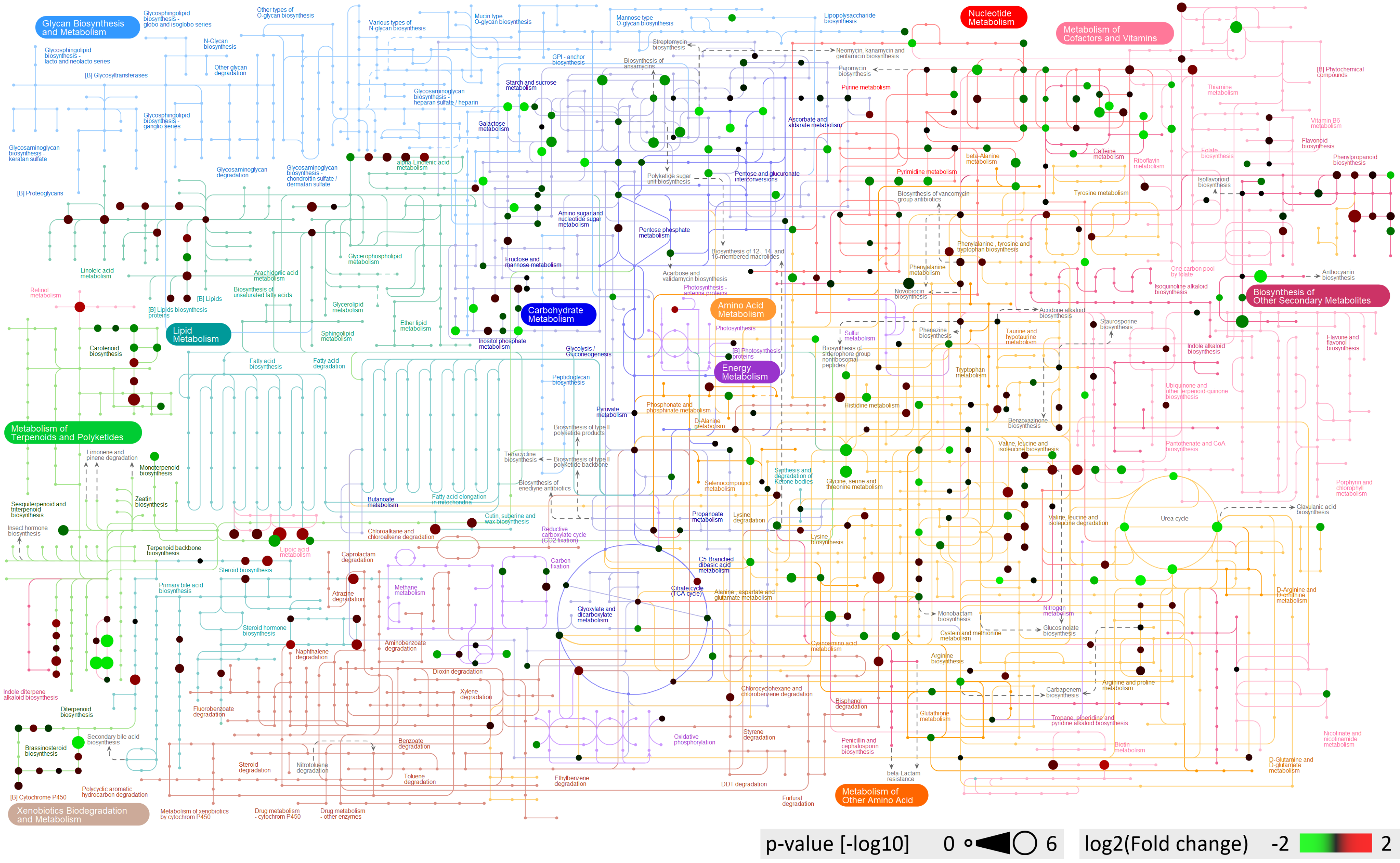

Relative changes in metabolite levels in the Arabidopsis mutant *thiC* (**a**) or wild type (**b**) plants upon supplementation with 1.5 uM thiamine. Polar metabolite extracts were analysed by non-targeted metabolomics. Peaks were putatively associated to chemical formulas and metabolites by accurate mass and isotopic patterns. Dot size indicates statistical significance obtained by a two-tailed heteroscedastic *t*-test adjusted for multiple hypothesis testing by the Benjamini-Hochberg procedure. Dot colour indicates the log2 transformed fold-change of the means of either thiamine-supplemented or control plants. Red dots indicate metabolites that are higher in the presence of thiamine. In the *thiC* mutant, thiamine supplementation causes a drastic increase of metabolites all over primary metabolism.

Supplementary Figure 2

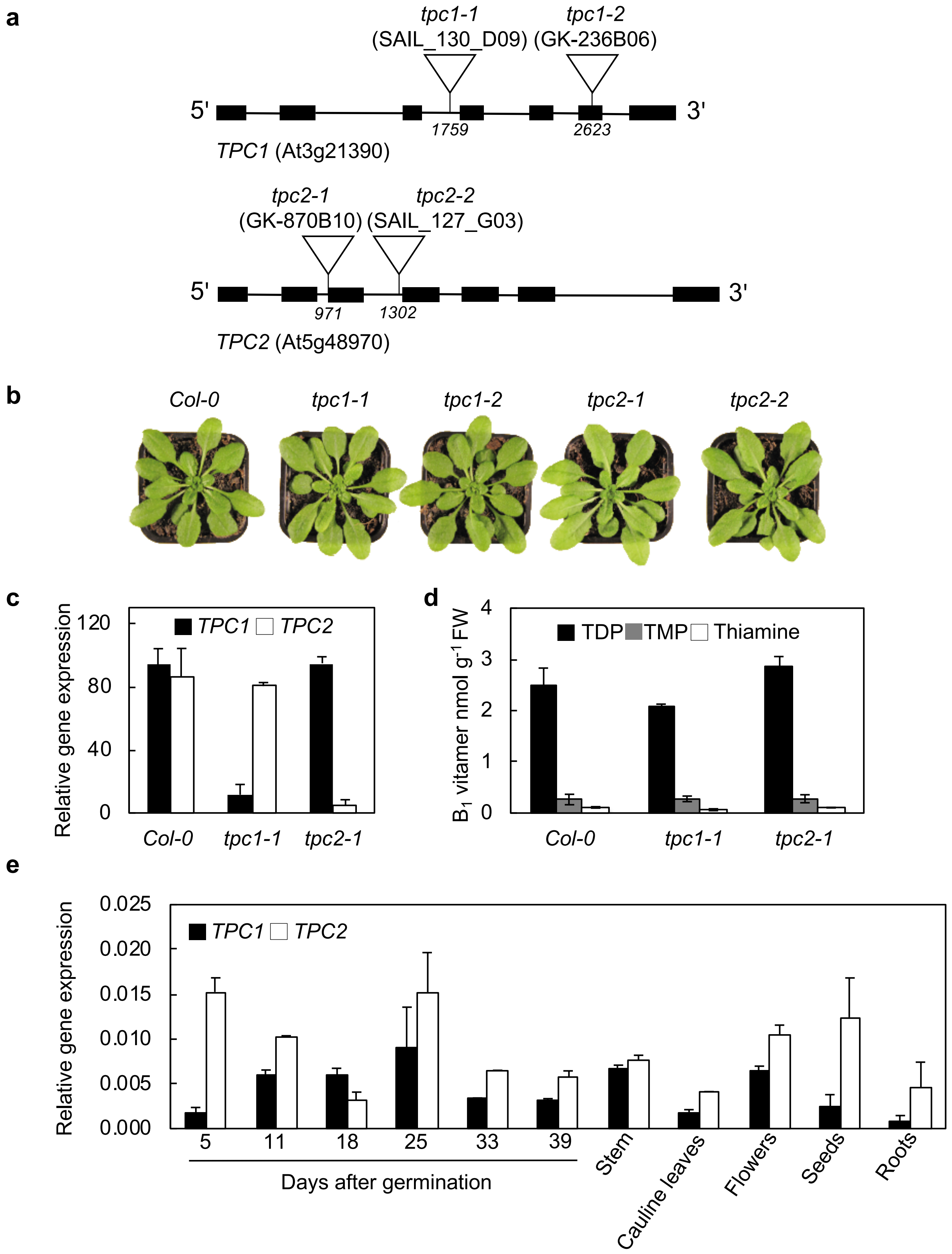

TPC1 and *TPC2* are redundant in Arabidopsis. **a**, Gene models of *TPC1* and *TPC2* with exons depicted as black bars and introns as lines. The location of the T-DNA insertion in SAIL_130_D09 (*tpc1-1*), GK-236B06 (*tpc1-2*), GK-870B10 (*tpc2-1*) and SAIL_127_G03 (*tpc2-2*) are as depicted and were confirmed by genotyping and sequencing. **b**, Representative photographs of 21-day old *tpc* lines compared to wild-type (Col-0), showing no morphological defects during leaf development. **c**, Quantitative analysis of *TPC1* or *TPC2* expression in mutant lines relative to wild type (Col-0) from rosette leaves of 21-day old plants. The data are the average of three independent biological replicates with error bars representing SD. **d**, Thiamine, thiamine monophosphate (TMP) or thiamine diphosphate (TDP) content of lines as indicated, as determined by HPLC from rosette leaves of 21-day old plants. No statistically significant differences were observed for any of the vitamers across the lines. **e**, Tissue expression analysis of *TPC1* and *TPC2* transcript levels by quantitative real-time RT-PCR relative to *ubiquitin carrier protein 21* (*UBC21*, At5g53300). Error bars represent the SD of three biological replicates with three technical repeats. In all cases, plants were grown on soil under a 16-hour photoperiod (120 umol photons.m^{-2} s^{-1}) at 22°C and 8 hours of darkness at 18°C.

Supplementary Figure 3

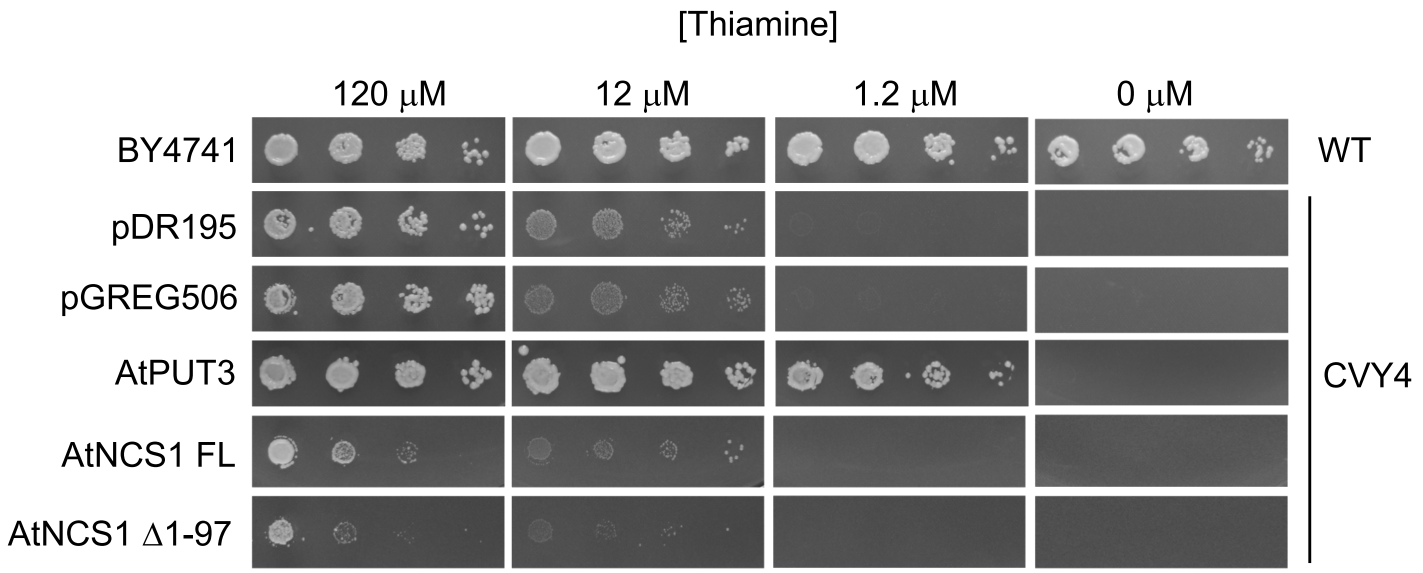

Growth of *Saccharomyces cerevisiae* (yeast) strain CVY4 (MATa *his3Δ1 leu2Δ0 lys2Δ0 ura3Δ0 thi4Δ::his5*$^{+}$ *thi7Δ::KanMX4 thi71Δ::LEU2 thi72Δ::LYS2*) that is impaired in biosynthesis and transport of thiamine in the presence of either the empty vector pDR195, or the empty vector pGREG506, or *Arabidopsis thaliana* PUT3 (thiamine transporter, AtPUT3 in pGREG506), or *Arabidopsis thaliana* NCS1 full length in pDR195 (AtNCS1 FL) or AtNCS1 in pGREG506 missing residues 1-97 from the N terminus (AtNCS1 Δ1-97). Pictures were captured after 3 days of growth on synthetic dextrose medium supplemented with the indicated thiamine concentrations. The individual rows of yeast growth represent 10-fold serial dilutions of the strains as indicated.

Supplementary Figure 4

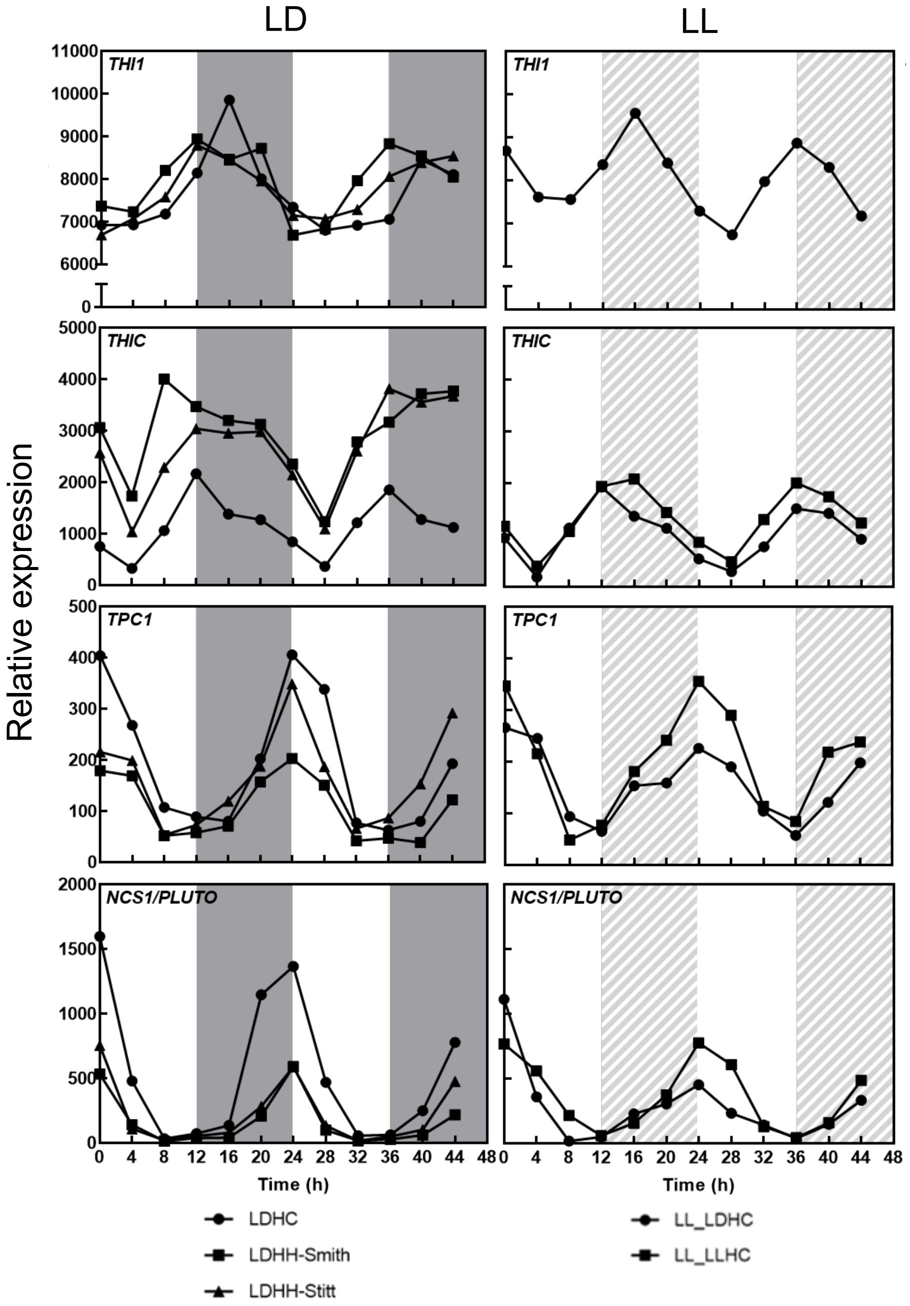

Transcript abundance of selected TDP biosynthesis and transport genes under equinoctial (12 hour light, 12 hour dark; LD) or continuous light (LL) conditions taken from Mockler TC et al 2007. In the case of LD, three sets of conditions were used to extract data: (LDHC), 7-day-old Col-0 seedling grown on ½ MS agar no sucrose; 12 hour light 100 µE at 22˚C, 12 h dark at 12˚C; (LDHH-Smith), 35-day-old Col-0 leaf grown on soil, 12 hour light 130 µE at 22˚C and 12 h dark at 22˚C; (LDHH-Stitt), 29-day-old Col-0 leaf grown on soil, 12 hour light 180 µE at 20˚C, 12 hour dark at 20˚C. In the case of LL, (LL-LDHC) two sets of conditions were used to extract data, 9-day-old Col-0 seedling grown on ½ MS agar no sucrose; 100 µE light at 22˚C; (LL-LLHC) reported to be the same as LL-LDHC.

Reference:

Mockler, T. C., Michael, T. P., Priest, H. D,, Shen, R., Sullivan, C. M., et al THE DIURNAL PROJECT: Diurnal and circadian expression profiling, model-based pattern matching and promoter analysis. *Cold Spring Harb. Symp. Quant. Biol.* **72**, 353-363 (2007)

Supplementary Tables

Supplementary Table 1. Ratio of antibiotic resistant (^{R}) to sensitive (^{S}) ($Sulf^R$:$Sulf^S$, $Basta^R$:$Basta^S$, $Sulf^R$ $Basta^R$:$Sulf^S$ $Basta^S$) for the F1 progeny of self-fertilized *Arabidopsis TPC1*/*tpc1.1* x *TPC2*/*tpc2.1* plants. Chi-squared (χ^2) p-values were calculated for the hypothesized segregation ratio from the tested ratio ($p < 0.05$, represented as *; not significant, ns). Sulf, refers to sulfadiazine; Basta, refers to phosphinotricin.

	$Sulf^R$	$Sulf^s$	Ratio tested	χ^2 p-value	$Basta^R$	$Basta^s$	Ratio tested	χ^2 p-value	$Sulf^R$ $Basta^R$	$Sulf^S$ $Basta^S$	Ratio tested	χ^2 p-value
tpc1.1 x tpc2.1	462	224	3:1<br>2:1	*<br>ns	626	206	3:1	ns	577	425	9:7	ns
tpc2.1 x tpc1.1	416	227	3:1<br>2:1	*<br>ns	430	129	3:1	ns	348	314	9:7	ns

Supplementary Table 2. List of oligonucleotide sequences used in this study.

YFP refers to Yellow Fluorescent Protein. IR and IS are Intron Retained and Intron Spliced, respectively. Sc refers to primers used for yeast (*Saccharomyces cerevisiae*) studies.

Gene target	Locus ID	Direction	Sequence (5′ – 3′)
CCA1	At2g46830	Forward	GCACTTTCCGCGAGTTCTTG
CCA1	At2g46830	Reverse	TGACTCCTTTCTTACCCTGTTATTCTG
LUC-IR	-	Forward	CCGTATAAGTCGACAAGGCAAAT
LUC-IS	-	Reverse	CAAGCACCCCAACAGTTTGT
NCS1qPCR	At5g03555	Forward	CGTGTTTCAGCCATGGAGATTGC
NCS1qPCR	At5g03555	Reverse	AAGCGCTGAGTACCCTATGAGC
NCS1-YFP	At5g03555	Forward	AAAAAGCAGGCTTGATGGTCTCCAATTGCTTAAG
NCS1-YFP	At5g03555	Reverse	AGAAAGCTGGGTACAAAAGCGGATGTGAAG
NCS1 FL (Sc)	At5g03555	Forward	GAATTCGATATCAAGCTTATCGATACCGTCGACAATGGTCTCCA ATTGCTTAAG CC
NCS1 FL (Sc)	At5g03555	Reverse	GCGTGACATAACTAATTACATGACTCGAGGTCGACTTACAAAAGCGGATGTGAAGAAG
NCS1 Δ97 (Sc)	At5g03555	Forward	GAATTCGATATCAAGCTTATCGATACCGTCGACAATGACCGGCTCAGAAATTAATGAC
TH1	At1g22940	Forward	TGTTAAAGGTGGTGATCTTCCTG
TH1	At1g22940	Reverse	TCTTGTAGCTATGCGAGGAGAAC
TH2	At5g32470	Forward	GGGGTCTCATTTGTGCCTTTG
TH2	At5g32470	Reverse	CTCCCATCCAAGAGCGAATG
THI1	At5g54770	Forward	AATCTGTTAGTCCTGGTGGTGGT
THI1	At5g54770	Reverse	CAATCTCGTCAAGGAACAAGTG
THIC	At2g29630	Forward	CCATCTTTTGAAGAATGCTTTCCT
THIC	At2g29630	Reverse	GAACACGACGAAAGGGAACTTT
THIC-IR	At2g29630	Forward	GCCTGTTGGACTATACCTGGATAAA
THIC-IR	At2g29630	Reverse	TGACTCAAATGAACAGACAACATAGATAGTT
THIC-IS	At2g29630	Forward	CTTGGTGCCTGTTGGACTATACC
THIC-IS	At2g29630	Reverse	TCAGGTTCAAAGGGACTTTCTCA
TPK1	At1g02880	Forward	TGCCTCATCCAACTCCTT
TPK1	At1g02880	Reverse	GTGTTGCTGAGATCCCATTG
TPK2	At2g44750	Forward	GAGAGTTCCAGACTCCAGAT

TPK2	At2g44750	Reverse	TGGAAGGAGTTGGATGAGAC
TPC1	At3g21390	Forward	TATGCTGGTCTGCAGTTTG
TPC1	At3g21390	Reverse	GCTTGATGAAGAAGATCGGT
TPC2	At5g48970	Forward	AATATTGGCCTCACAAGGG
TPC2	At5g48970	Reverse	CCTCTTATACCTCGAGACTG
UBC21	At5g25760	Forward	TAGCATTGATGGCTCATCCTGA
UBC21	At5g25760	Reverse	TTGTGCCATTGAATTGAACCC
