# Supplementary material for: The coenzyme thiamine diphosphate displays a daily rhythm in the Arabidopsis nucleus
Source: Commun Biol. 2020 May 5;3:209. doi: 10.1038/s42003-020-0927-z (PMC7200797; doi:10.1038/s42003-020-0927-z)
Supplement: Supplementary file 4 — Description of Additional Supplementary Files [file 42003_2020_927_MOESM4_ESM.pdf]

Description of Additional Supplementary Files

File Name: Supplementary Data 1
Description: contains the source data underlying plots shown in the main figures of this study.
